# SARS-CoV-2 exposure in deer increases with use of human-modified habitat

**DOI:** 10.1098/rsbl.2026.0410

**Published:** 2026-11-04

**Authors:** Tyler J. Garwood, Jeremy Alder, Sara Hathaway, George Wittemyer, Guillaume Bastille-Rousseau, Tadao Kishimoto, Michael Egan, Travis Gallo, Jennifer M. Mullinax, Amira A. Roess, Taylor Anderson, Tiffany M. Wolf, James D. Forester, Jennifer L. Malmberg, Marnee Roundtree, Georgia C. Titcomb, Rebecca M. Windell, Maria A. Diuk-Wasser, Laura D. Plimpton, Meredith C. VanAcker, Daniel M. Grove, Justin R. Kosiewska, Dailee L. Metts, Cameron Mitchell, Lisa I. Muller, Mark Q. Wilber, Jacob D. Wyrick, Kezia R. Manlove, Megan Morrison, Aung N. Chan, Jennifer Høy-Petersen, Roderick B. Gagne, W. David Walter, Alec Baker, Jeffrey C. Chandler, Grete Wilson-Henjum, Kim M. Pepin, Meggan E. Craft

**Affiliations:** ^1^ Department of Ecology, Evolution, and Behavior, University of Minnesota, St Paul, MN, USA; ^2^ Department of Veterinary Population Medicine, University of Minnesota, St Paul, MN, USA; ^3^ Department of Fisheries, Wildlife, and Conservation Biology, University of Minnesota, St Paul, MN, USA; ^4^ Department of Fish, Wildlife and Conservation Biology, Colorado State University, Fort Collins, CO, USA; ^5^ Center for Wildlife Sustainability Research, Southern Illinois University, Carbondale, IL, USA; ^6^ Department of Environmental Sciences and Technology, University of Maryland, College Park, MD, USA; ^7^ Department of Global and Community Health, George Mason University, Fairfax, VA, USA; ^8^ Department of Geography and Geoinformation Science, George Mason University, Fairfax, VA, USA; ^9^ National Wildlife Research Center, Wildlife Services, Animal and Plant Health Inspection Service, United States Department of Agriculture, Fort Collins, CO, USA; ^10^ Nebraska Game and Parks Commission, Alliance, NE, USA; ^11^ Department of Ecology, Evolution, and Environmental Biology, Columbia University, New York, NY, USA; ^12^ Department of Evolution, Ecology, and Organismal Biology, University of California Riverside, Riverside, CA, USA; ^13^ School of Natural Resources, University of Tennessee Institute of Agriculture, Knoxville, TN, USA; ^14^ Department of Wildland Resources and Ecology Center, Utah State University, Logan, UT, USA; ^15^ Department of Pathobiology, Wildlife Futures Program, University of Pennsylvania School of Veterinary Medicine, Kennett Square, PA, USA; ^16^ Department of Veterinary Microbiology and Pathology, College of Veterinary Medicine, Washington State University, Pullman, WA, USA; ^17^ Pennsylvania Cooperative Fish and Wildlife Research Unit, The Pennsylvania State University, University Park, PA, USA

**Keywords:** One Health, mule deer, spillover, human–wildlife interface, zoonosis, habitat use, animal movement, disease transmission, white-tailed deer

## Abstract

Deer can transmit severe acute respiratory syndrome coronavirus 2 (SARS-CoV-2) to humans and harbour human-extinct variants. Despite this public health threat, ecological mechanisms driving exposure patterns in deer populations remain poorly understood. Our objective was to identify behavioural risk factors by linking serostatus of individual deer to their movement and space use using multilevel logistic regression. We examined serosurveillance data of white-tailed (*Odocoileus virginianus*) and mule deer (*O. hemionus*) wearing global positioning system devices across an urban–rural gradient in the United States (1151 individuals, 20 sites, 8 states). Seropositivity was positively associated with average human footprint index and negatively associated with average SARS-CoV-2 human wastewater concentration. Seropositivity was not associated with time spent in GAP-modelled deer habitat. Together, these findings provide evidence that human-to-deer transmission could be a primary source of deer SARS-CoV-2 exposure and deer populations may struggle to maintain transmission without repeated spillover from humans. Integrating human and environmental indices with movement behaviours and space use can thus clarify pathways that facilitate cross-species transmission in wildlife.

## 1. Introduction

Variation in host movement and space use can drive disease transmission dynamics [1–3]. For emerging diseases with unresolved ecologies, understanding the association between movement or space use and pathogen infection or exposure can shed insight on hypothesized transmission routes and improve modelling efforts [4–6]. However, these data are difficult to acquire, leaving individual-level movement and space use drivers of infection and exposure poorly understood in important disease systems.

Severe acute respiratory syndrome coronavirus 2 (SARS-CoV-2) caused 1.2 million excess human deaths in the years following emergence and continues to evolve [7–9]. The evolution of new variants or maintenance of human-extinct variants in animals, followed by spillback to humans, could result in re-emergence [10]. Because of this potential public health threat, understanding the factors influencing SARS-CoV-2 infection and exposure at the human–animal interface remains a priority [11].

Mule deer (*Odocoileus hemionus*) and white-tailed deer (*O. virginianus*; ‘deer’ or MD/WTD) can be infected with SARS-CoV-2 and transmit it to conspecifics or humans [12–14]. Deer can carry human-extinct variants and provide a host environment for novel genetic variation [15,16], indicating that they could maintain SARS-CoV-2, provide a new host environment for selection and facilitate variant spillback into humans. Therefore, understanding where and how deer contract SARS-CoV-2 is vital to risk assessment.

SARS-CoV-2 is more likely to mutate and be maintained in deer populations if the virus is sustained independently of repeated infections from humans [17,18]. One way to explore this is to assess the association between space use and infection or exposure status, particularly as it relates to human- or deer-dense landscapes. Higher SARS-CoV-2 exposure or positivity rates among synanthropic deer than those in higher deer density areas would suggest human spillover is a primary infection driver. Certain movement traits, such as ranging further in human-modified habitats, could also increase infection probability [19–21].

Previous national SARS-CoV-2 studies of wild deer relied on post-mortem sampling [13,15] and provided limited inference about how individual movement and space use influence deer infection patterns. Studies that track individual locations through time are better suited to estimate movement behaviours and space use and relate them to disease status [22]. A previous study used WTD post-mortem samples to explore the effects of individual traits and county-level environmental risk factors on SARS-CoV-2 infection prevalence and found that human population density had a stronger positive effect on prevalence than available deer habitat, although both were positively associated with infection [23]. That county-level study indicated human spillover was a likely infection driver in deer, but the findings were susceptible to the ecological fallacy [24]. Hence, next steps would be to analyse individual-level space use metrics.

Our objective was to examine effects of deer movement and space use on SARS-CoV-2 exposure in deer. We implemented a cohort study design with antibody testing and global positioning system (GPS) collar tracking at sites across an urban–rural gradient. We used multilevel logistic regression to explore how human infection pressure, deer movement behaviours and deer space use variables were associated with seropositivity, while also accounting for deer demographic traits and study design. We predicted that deer who both spent more time in human development and moved at greater speeds would encounter more infected people and were more likely to be exposed.

## 2. Material and methods

### (a) Capture, disease sampling and collaring

Capture, disease testing and collaring methods were described in Pepin *et al.* [22]. Briefly, we captured deer at 20 sites covering an urban–rural gradient in the United States between 2 December 2022 and 10 April 2025 (figure 1; sample sizes in supplementary material, table S1). Capture methods included helicopters, ground darting, drop nets and clover traps (Institutional Animal Care and Use Committee protocol numbers in supplementary material). Upon capture, we obtained nasal and oropharyngeal swabs, which we tested for active SARS-CoV-2 infections via real-time polymerase chain reaction (PCR) with reverse transcription. Actively infected deer were defined by a cycle quantification value below 38 for either swab. Additionally, we collected blood to determine binary exposure status via surrogate virus neutralization tests. ‘Seropositive’ testing events were defined as one positive result (more than 30% inhibition for wild type, more than 40% for omicron) from any of the possible sample (Nobuto or serum)/strain type (wild type or omicron) combinations for a given individual (inhibition thresholds determined in Hewitt *et al.* [26]). Sample handling and laboratory procedure details are in the supplementary material. For this analysis, we used the first sample set taken per deer.

**Figure 1.**
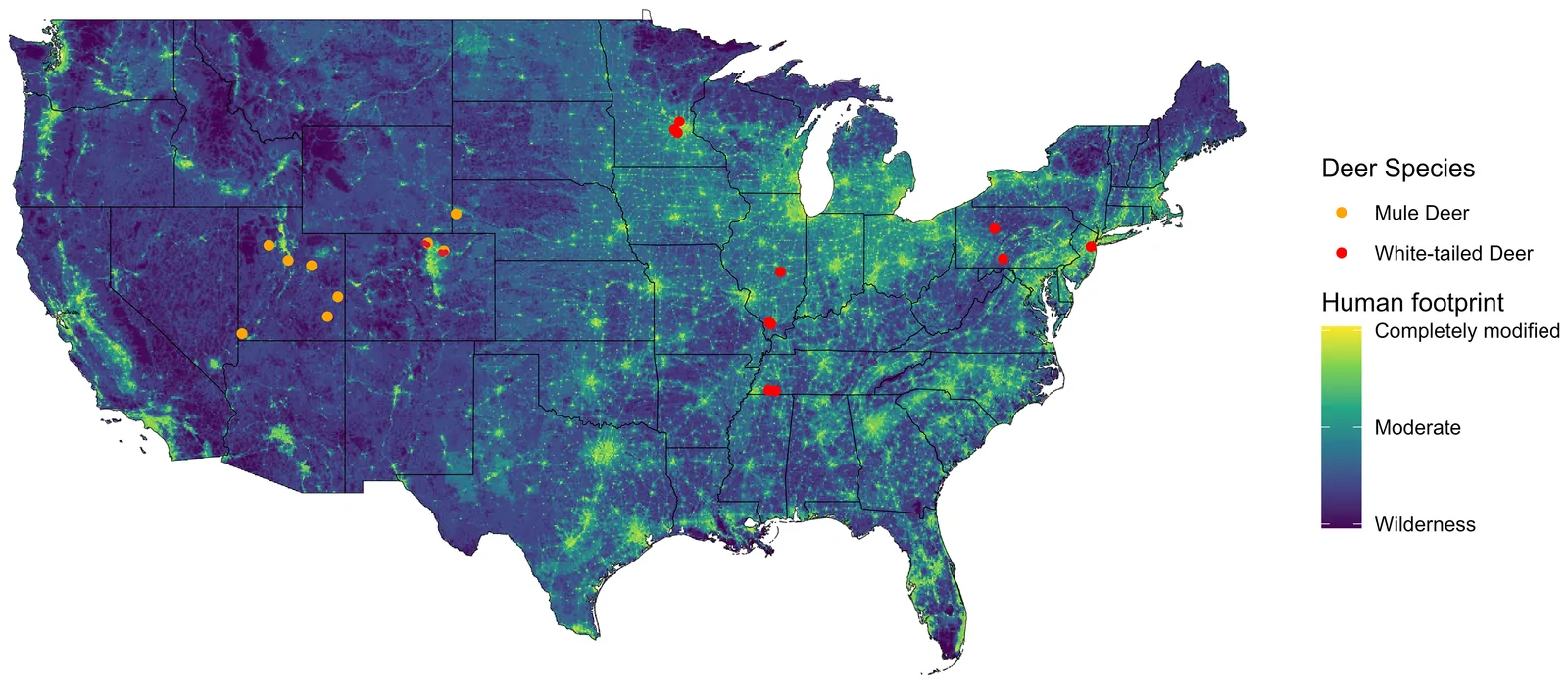
Study site locations across the United States in relation to the HFI, a metric that measures human pressure through combining layers describing pasture, roads, railways, population density, nighttime light, built environment and cropland [25].

We placed GPS collars on each deer and determined species, sex and age class (juvenile less than 1.5 years old, adult more than 1.5 years old [27]). Collars transmitted locations to satellites at a variety of intervals (summarized in supplementary material, table S2). This wide range of intervals was necessary because higher resolution location data (15- and 30-minute intervals) were stored on some collars, and most collars were still deployed at the time of writing.

### (b) GPS data cleaning and covariate extraction

We analysed location data from 2 December 2022 to 10 May 2025. We removed locations for the 3 days following capture, maintained a temporal duplicate threshold of less than or equal to 10 minutes between locations, and removed locations that indicated unlikely speeds or round trips (more than 3 km h^–1^, epsilon = 10 in the R package ‘amt’ function flag_roundtrips [28]).

For habitat covariates, we extracted National Land Cover Dataset classification (NLCD) [29], human population density [30], human footprint index (HFI) [25] and GAP-modelled deer habitat [25,31] at each location in the two-, four- and six-month time windows after capture (R package ‘terra’ [32]). We extracted these layers from a variety of years owing to data availability (table 1) [33–40]. We chose post-capture time window lengths to balance temporal proximity to the test date with biological relevance given that antibodies can persist for 13 months [41]. We later tested the importance of this choice via sensitivity analysis. We had GPS data post-capture and assumed that post-capture movements reflect space use in the 13 months pre-testing when exposure could have happened. This assumption is commonly made in studies blending disease prevalence and host movement data, including those examining WTD and MD [42–45]. Individuals captured within two, four or six months of the end of the study were excluded from the respective dataset. We also excluded deer that died within two weeks of capture [46] but included those that died before the end of the respective time window to avoid biasing results towards healthy survivors. Summary space use variables were calculated at the individual level (supplementary material). We standardized average human population density within each site via z-score; this variable therefore captures within-site differences between individuals rather than study-wide differences. Finally, we calculated and standardized county-level human wastewater concentrations of SARS-CoV-2 from the Center for Disease Control National Wastewater Surveillance System [47] in the 13 months before testing for each individual. Where county data were not available, we took data from the nearest county with similar human demographic traits.

**Table 1.** The variables, along with corresponding data sources and hypotheses, used to model SARS-CoV-2 seropositivity in deer.

covariate	general ecological process	data source/year/resolution in m	overlapping proxy within ecological process?^a^	hypothesis driving inclusion
***main effects***				
sex	deer demographic traits	collected at capture	no	males are more likely to be seropositive owing to testosterone being immunosuppressive [33]
species	deer demographic traits	collected at capture	no	WTD are more likely to be seropositive because of their preference for edge habitat [34] that may be near various sources of infection (domestic animals, fomites, humans etc.)
age	deer demographic traits	collected at capture	no	juveniles are less likely to be seropositive because they have had less time to acquire exposure [35,36]
median speed	movement behaviours	measured via collars	no	deer with a higher median speed might have higher rates of SARS-CoV-2 exposure because of an increase in encounter rates [37]
standardized average maximum 6-day range	movement behaviours	measured via collars	no	deer with a higher standardized average maximum 6-day range would be more likely to be seropositive because they may encounter more infection sources [38]
proportion of locations in development	time spent near humans or human-modified landscapes	USGS National Land Cover Database/2023/30	yes	deer that had a high proportion of locations in development would be more likely to be seropositive owing to increased exposure to humans, pets and fomites
standardized average human population density	time spent near humans or human-modified landscapes	NASA Socioeconomic Data and Applications Centre/2020/100	yes	deer that experienced high standardized human population density would be more likely to be seropositive owing to increased exposure to humans, pets and fomites
average HFI	time spent near humans or human-modified landscapes	Mu *et al.* Scientific Data, 2022/2018/1000	yes	deer that experienced a high HFI would be more likely to be seropositive owing to increased exposure to humans, pets and fomites
proportion of locations in GAP-modelled deer habitat	time spent in areas of high deer activity	USGS GAP Analysis Project/2001/30	yes	deer that spent more time in areas of high deer activity would be more likely to be seropositive because they were more likely to have increased contact with other deer. Here, in lieu of national deer density layers and to align with the previous county-level study of SARS-CoV-2 prevalence [23], we assume that ‘proportion of locations in GAP-modelled deer habitat’ is a reasonable proxy for elevated deer density. Previous studies have found that habitat suitability is strongly correlated with deer density [39]
standardized average SARS-CoV-2 human wastewater concentration	human infection pressure	CDC National Wastewater Surveillance System/2026/county	no	high relative active infections in humans in the months before testing leads to a higher probability of deer infection via interactions with infected fomites, human contact or domestic animals
biological year	sampling design	collected at capture (1 June–31 May)	no	at some sites, year-specific environmental conditions like snow cover and temperature changed drastically between years, which could plausibly alter a deer's use of development (i.e. more likely to be susceptible to feeding during heavy snow)—and consequently increase their likelihood of SARS-CoV-2 exposure in a given year
***interactions***				
proportion of locations in forest × species	time spent in areas of high deer activity	USGS National Land Cover Database/2023/30	yes	deer that spent more time in areas of high deer activity would be more likely to be seropositive because they were more likely to have increased contact with other deer. Here, in lieu of national deer density layers, we assume that WTD preferentially use forest and therefore experience higher deer densities and potentially more SARS-CoV-2 exposure there, whereas MD would avoid forest and similarly experience higher densities of other MD in those areas
average HFI × standardized average human wastewater concentration	human infection pressure	Mu *et al.* Scientific Data, 2022/2018/1000; collected at capture	no	spending more time in human habitat is more strongly associated with deer seropositivity when human SCV2 rates are relatively high
***random effect***				
site	sampling design	collected at capture	no	N/A

^a^That is, variables considered in ‘tier 1’ of the modelling process.

We calculated movement variables across post-capture windows. We calculated maximum distances between a deer's locations for each six-day period and took the average to measure how far a deer ranged during the SARS-CoV-2 infectious period [48]. We then standardized (*z*-score) the average maximum distance between locations by site. We calculated median speed (m h^–1^) in the R package ‘amt’ [28]. Because this metric depends on the time interval between locations [49,50], we standardized to a common fix rate (270 minutes) before summarization via *k*-steps normalization to avoid discarding location data collected at more frequent intervals (*sensu* ‘multi-step’ approach [51]; details in supplementary material).

### (c) Modelling

Our covariates were sex, species, age class, median speed, standardized average maximum six day range, proportion of locations in development (NLCD classes 21–24), standardized average human population density, average HFI (metric ranging from 0 to 50 that combines spatial data describing pasture, roads, railways, human population density, navigable waterways, nighttime light, built environment and cropland in a single layer [25]), standardized average SARS-CoV-2 human wastewater concentration, proportion of locations in GAP-modelled deer habitat [25,31], and biological year (1 June–31 May). Interactions are listed in table 1. We tested correlations between predictor variables using Pearson's correlation coefficient (less than 0.7, the R package ‘corrr’ [52,53]) and for collinearity via variance inflation factor (less than 5, the R package ‘performance’ [54,55]).

We considered covariates through the lens of six ecological processes associated with deer seropositivity probability; deer demographic traits, movement behaviours, local human infection pressure, time spent near humans or human-modified landscapes, time spent in areas of high deer activity and sampling design could all account for the seropositivity patterns we observed (table 1 gives specific covariate categorizations and hypotheses).

Several covariates overlapped in the process they measured: proportion of locations in development, standardized mean human population density and average HFI each index human activity. Likewise, proportion of locations in GAP-modelled deer habitat and proportion of locations in forest × species (i.e. species-specific responses to forest cover) represent deer activity. To preserve interpretability, we did not include multiple proxies for the same phenomenon in the same model. Instead, we used a two-tier information–theoretic model selection approach. In tier 1, for each phenomenon (human activity and deer activity), we fitted otherwise identical baseline models that differed only in which proxy variable was included (table 2). We compared models using corrected Akaike's information criterion [56] (AICc; calculated in AICcmodavg [57]) and retained for subsequent analyses the best proxy for each phenomenon (*sensu* [58]). In tier 2, we built a candidate model set reflecting our hypotheses about SARS-CoV-2 exposure. Models within ΔAICc ≤ 2 were competitive, and we favoured the most parsimonious model with the lowest AICc [59,60]. All models were multilevel logistic regressions (the R package ‘lme4’ [61]) that included biological year, sex, age class and species as fixed variables and site as a random intercept [62] to account for physiological and study design sources of variation. We used seropositivity status of individuals as the response variable. We always included movement variables together to characterize the overall movement intensity of an individual. We performed model selection on the dataset with covariates extracted from the four-month post-capture window.

**Table 2.** Models considered for explaining SARS-CoV-2 seropositivity in deer, ordered by ΔAICc.

	parameter number	weight	AICc	ΔAICc
**tier 1: selecting between covariates that represent overlapping ecological constructs**				
*models examining the best covariate for deer habitat*				
biological year + sex + age class + prop. forest * species	9	0.705	826.62	0
biological year + sex + age class + species + prop. GAP deer habitat	8	0.295	828.36	1.74
*models examining human activity*				
biological year + sex + age class + species + average HFI	8	0.960	814.09	0
biological year + sex + age class + species + prop. development	8	0.036	820.67	6.58
biological year + sex + age class + species + st. av. human population density	8	0.004	824.98	10.89
**tier 2: selecting between *a priori* candidate models using best terms for a given ecological construct from tier 1**				
*candidate models*				
biological year + age class + sex + species + av. HFI * st. av. human wastewater concentration	10	0.449	809.40	0
biological year + age class + sex + species + av. HFI + st. av. human wastewater concentration	9	0.297	810.23	0.83
biological year + age class + sex + species + av. HFI * st. av. human wastewater concentration + prop. GAP deer habitat	11	0.164	811.42	2.02
biological year + age class + sex + species + av. HFI	8	0.043	814.09	4.69
biological year + age class + sex + species + av. HFI * st. av. human wastewater concentration + prop. GAP deer habitat + median speed + st. av. max. 6-day range	13	0.021	815.48	6.08
biological year + age class + sex + species + av. HFI + prop. GAP deer habitat	9	0.017	815.95	6.55
biological year + age class + sex + species + av. HFI + median speed + st. av. max. 6-day range	10	0.006	818.05	8.65
biological year + age class + sex + species + av. HFI + prop. GAP deer habitat + median speed + st. av. max. 6-day range	11	0.002	819.90	10.50
biological year + age class + sex + species + st. av. human wastewater concentration + prop. GAP deer habitat	9	0	824.88	15.48
biological year + age class + sex + species + st. av. human wastewater concentration + median speed + st. av. max. 6-day range	10	0	826.24	16.84
biological year + age class + sex + species	7	0	826.35	16.95
biological year + age class + sex + species + prop. GAP deer habitat	8	0	828.36	18.96
biological year + age class + sex + species + median speed + st. av. max. 6-day range	9	0	829.52	20.12
biological year + age class + sex + species + prop. GAP deer habitat + median speed + st. av. max. 6-day range	10	0	831.51	22.11

Because the proportion of locations in human development, standardized average human population density and average HFI are human activity proxies, and the proportion of locations in GAP-modelled deer habitat and the proportion of locations in forest * species for deer habitat are deer activity proxies, we avoided including multiple proxies for each phenomenon in the same model. Hence, we used a two-tier information–theoretic workflow: (i) fitted otherwise identical models that differed by a single proxy and retained the best proxy; (ii) built models with the chosen proxies. Models within ΔAICc ≤ 2 were considered competitive; ties were resolved by parsimony.

To evaluate whether inference depended on the post-capture temporal window used, we refitted the top model using covariates calculated over two-, four- and six-month periods. We compared parameter estimates across windows to assess the stability of covariate effects. We then calculated AICc values for each window using only the subset of individuals present in all three datasets, allowing AICcs to be directly comparable. We selected the window with the lowest AICc for reporting top model results, which was refitted using the full dataset. We confirmed the assumption of uniformity in the randomized quantile residuals (the R package ‘dHARMA’ [63]). We additionally performed a leave-one-out (LOO) validation by site to confirm our results were not driven by a single site and assessed covariate stability across the candidate model set.

## 3. Results

### (a) Capture, disease testing and covariates

We captured, tested and collared 1151 deer (698 MD, 453 WTD; supplementary material, table S1) from 2 December 2022 to 6 January 2025 (capture timing and sample demographic summary by site in supplementary material, figure S1 and table S1). We observed 181 seropositive and 970 seronegative deer (16% seropositive), with variation between sites (figure 2a). Nine deer (approx. 0.5%) were infected with SARS-CoV-2. Our sample skewed towards adults (945 adults, 206 juveniles) and females (920 females, 231 males). We sampled 324 deer in the biological year 2022–2023, 670 in 2023–2024 and 157 in 2024–2025. On average, deer occupied landscapes with an HFI of 17 ± 10 (1 s.d.; high, 38.4 ± 1.3 in New York and low, 10.7 ± 5.5 in Utah), were exposed to a human wastewater concentration value of 197 691 490 ± 187 651 131 gene copies/person/day (high, 762 730 572 ± 2 554 045 in New York and low, 25 224 703 ± 27 184 277 in Pennsylvania), had median speeds of 52 ± 19 m h^–1^ (high, 84.1 ± 20.9 in Nebraska and low, 41.6 ± 13.3 in New York) and a mean 6-day range of 2440 ± 878 m (high, 3682 ± 592 in Nebraska and low, 1206 ± 210 in New York). Deer spent 29 ± 0.29% (high, 70 ± 18% in Pennsylvania and low: 1 ± 3% in Colorado) of their time within forested habitat and 85 ± 0.23% of their time in GAP-modelled deer habitat (high, 98 ± 3% in Minnesota and low, 31 ± 24% in Nebraska; covariates summarized in figure 2b; supplementary material, table S1). No strong correlations were detected among covariates (supplementary material, table S3).

**Figure 2.**
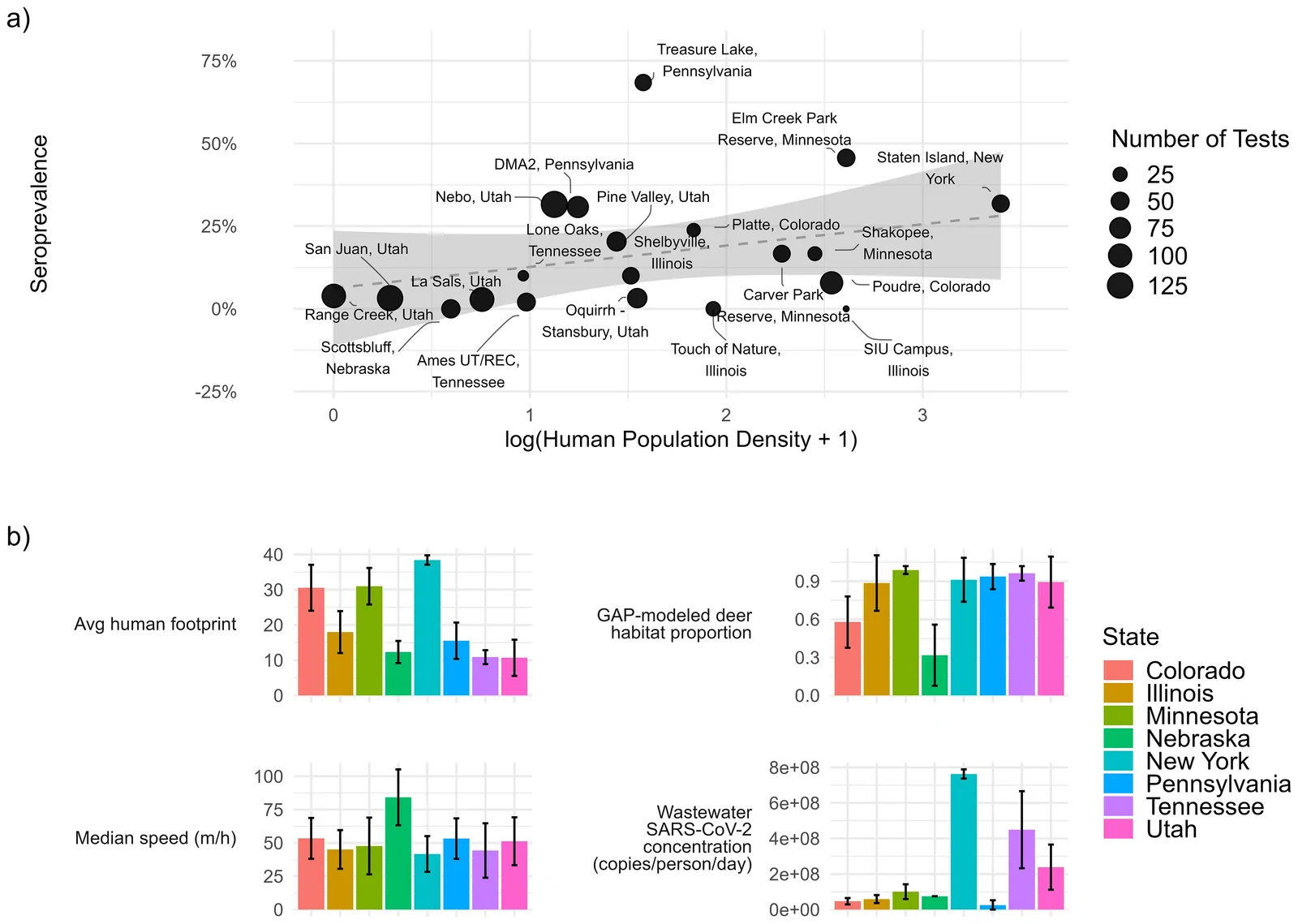
(a) The site-level relationship between human population density, calculated as the average human population density within the minimum convex polygon of all deer at a site, and deer SARS-CoV-2 seroprevalence based on testing between 2 December 2022–6 January 2025. (b) A summary of important covariates calculated over the two-month post-capture window by state. Error bars represent 1 s.d.

### (b) Modelling

Our top model was0

$$\begin{array}{c} \begin{array}{c} logit\left(Pr\left(Seropositive_{i}\right)\right)\;=\;\beta_{0}\\ +\beta_{1}\times species_{i}\;+\beta_{2}\times age\;class_{i}\;+\beta_{3}\times sex_{i}\;+\beta_{4}\times \;biological\;year\;2023-2024_{i}+\beta_{5}\\ \times \text{biological year}2024-2025_{i}\\ +\beta_{6}\times {\text{average human footprint index}}_{i}+\beta_{7}\\ \times {\text{standardized average human wastewater concentration}}_{i}\\ +\;u_{site\;\left[i\right]}, \end{array} \end{array}$$

where $\mathrm{logit}(Pr(\mathrm{Seropositiv}e_{i}))$ is the log-odds of a seropositive individual *i* and $u_{\mathrm{site}\;[i]}∼N\left(0,\;\sigma_{\mathrm{site}}^{2}\right)$ is the random intercept for the site where individual *i* was sampled. Models in the selection process were built with the full sample set (*n* = 1151). Variables positively associated with seropositivity were average HFI (*β*_6_ = 0.07, s.e. = 0.02, *p* < 0.05) and species (MD is baseline, *β*_1_ = 0.97, s.e. = 0.28, *p* = 0.05; figure 3). Lower seropositivity was associated with being tested in biological year 2023–2024 (2022–2023 is baseline, *β*_4_ = −0.48, s.e. = 0.29, *p* = 0.10) or in biological year 2024–2025 (*β*_5_ = –1.06, s.e. = 0.50, *p* < 0.05), being a male (female is baseline, *β*_3_ = −0.13, s.e. = 0.23, *p* = 0.58), higher standardized average human wastewater concentration (*β*_7_ = −0.28, s.e. = 0.12, *p* < 0.05) and being a juvenile (adult is baseline: *β*_2_ = −1.12, s.e. = 0.28, *p* < 0.05). Our only other competitive model included all top model terms with the addition of an interaction between average HFI and standardized average SARS-CoV-2 human wastewater concentration (ΔAICc = −0.83; table 2).

**Figure 3.**
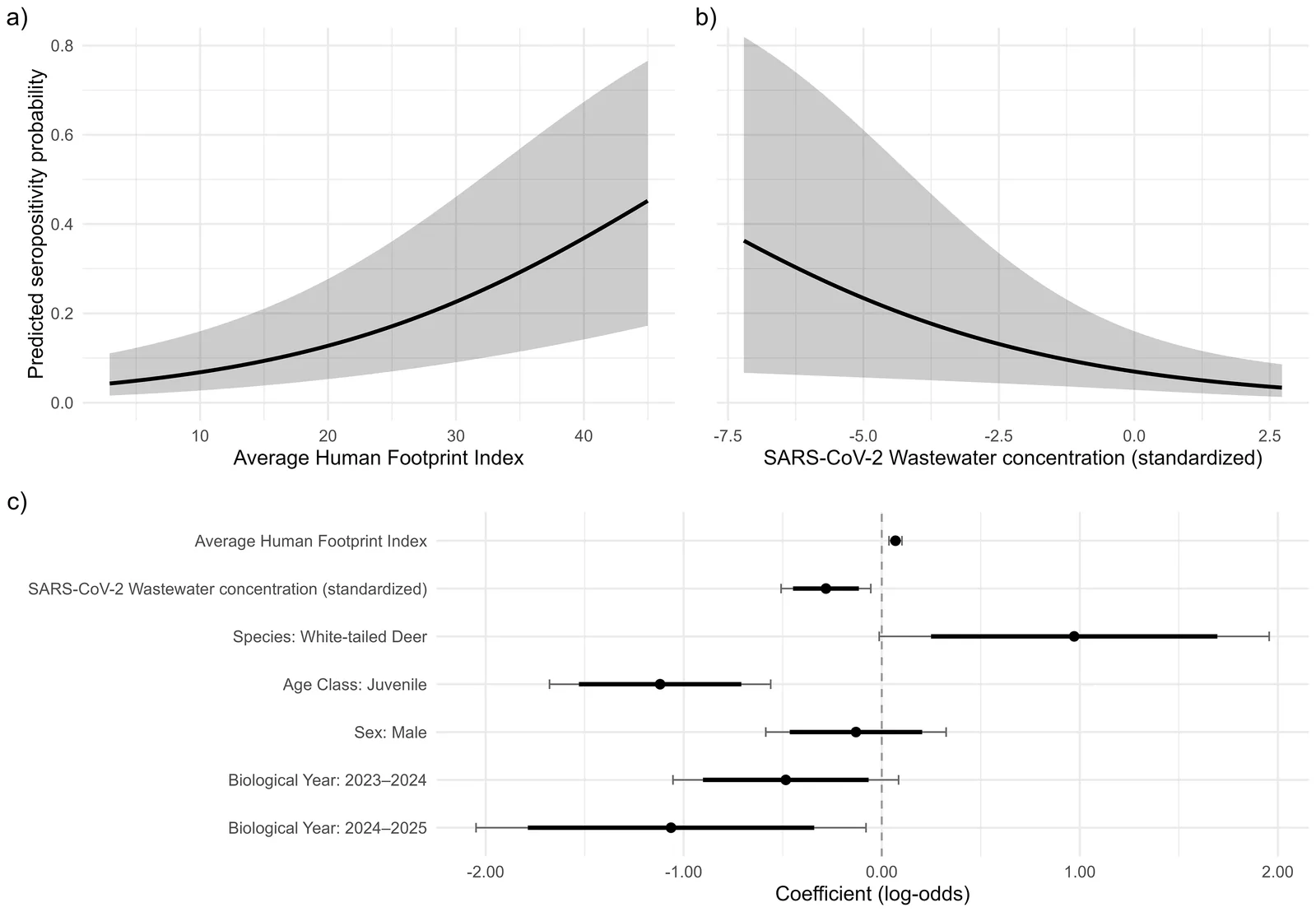
Predicted seropositivity probabilities for deer based on our top multilevel logistic regression model and the two-month window across the observed value range for (a) average HFI and (b) *z*-score standardized SARS-CoV-2 human wastewater concentration values. (c) The coefficient values and 85% (thick lines) and 95% confidence intervals (thin lines) for variables in our top model.

All coefficients were stable across LOO validation (supplementary material, table S4). Similarly, effect sizes and directions were consistent across the two-, four- and six-month post-capture time windows, as well as across candidate models (supplementary material, figures S2 and S3). We reported coefficient values from the two-month time window (*n* = 1115; supplementary material, figure S4).

## 4. Discussion

We leveraged movement and disease data from an urban–rural gradient to evaluate potential drivers of deer SARS-CoV-2 exposure. We found that deer using more human-modified habitats and in counties with lower average human SARS-CoV-2 wastewater concentrations had higher SARS-CoV-2 seropositivity. Exposure also varied significantly with age class, species and biological year.

Deer with higher proportions of locations in human-modified landscapes had significantly higher SARS-CoV-2 seropositivity (figure 3a), which is consistent with county-level associations between use of human-developed landscapes and infection [23]. However, our study diverges on the effect of spending more time in areas of high deer activity. Hewitt *et al.* reported a positive county-level association between GAP-modelled deer habitat and infection [23], but we found that the proportion of deer locations in GAP-modelled deer habitat had no association with seropositivity. The variable fix rates in our study prevented us from exploring whether deer–deer contact rate was associated with seropositivity, which is a factor worth exploring in future studies examining the role of deer–deer in SARS-CoV-2 transmission. Regardless, our finding suggests that circulation and maintenance within deer populations is less likely than previously thought, and repeated spillover from humans may be a driver of deer seropositivity rates. Modelling at the individual rather than population level allowed us to show that the deer using human-modified habitat were the ones more likely to be seropositive.

Average SARS-CoV-2 wastewater concentration was negatively associated with seropositivity (figure 3b), but this finding should be interpreted cautiously. Intuitively, higher SARS-CoV-2 infection pressure from humans should be positively associated with seropositivity in deer, especially given our finding that deer exposure is significantly associated with increased use of human-modified habitats. A potential explanation is that the wastewater concentration metric was calculated at the county level over the 13 months before testing, and is therefore susceptible to the same ecological fallacy as post-mortem deer studies [23]. Temporal aggregation over 13 months may have dampened the effect of human wastewater peaks. Similarly, because wastewater data are aggregated at the county level, the measured signal likely originated from treatment facilities serving a county's urban centre, whereas some of our capture sites were located in rural portions of the same county. A single county-level concentration therefore assigns identical human infection pressure to areas that differ substantially in human density, likely attenuating our ability to detect an association. This effect has been identified previously in landscape-scale analyses of surveillance data [64]. For example, larger cities have bigger outbreaks of SARS-CoV-2 [65], and proximity to larger city centres can translate to more cases [66,67]. With human caseload reporting continuing to decline, it is unlikely that finer-scale data to explore this relationship will become available at a national level [68].

We were surprised that deer that exhibited larger ranges and moved more quickly were not significantly associated with higher seropositivity (table 2; supplementary material, figure S3), as these traits could result in more contacts with infectious agents [21]. Biologically, it is plausible that spending time in human-modified areas results in greater transmission regardless of movement characteristics, especially if transmission occurs at discrete, high-risk locations and a deer's home range is centred on those point sources [69]. However, theoretical work informed by data from our system suggests that movement should influence transmission rates [70]. Our coarse and variable sampling interval prevented us from calculating finer scale movement metrics that may be more tightly associated with seropositivity. Ultimately, given that movement behaviours have previously been correlated with pathogen exposure in this system [70], we maintain that datasets with short, common fix rates may be better suited to explore this question and do not rule out the importance of variation in individual movement metrics on SARS-CoV-2 exposure in deer.

Despite high seroprevalence at some sites, we found minimal evidence that SARS-CoV-2 was actively circulating in most of our deer populations. We had nine PCR-positive tests despite sampling a wide environmental gradient. Our apparent infection prevalence (approx. 0.5%) is much lower than earlier studies, which had positivity rates as high as 82.5% in the autumn of 2020 [8] and 11.3–21.5% in the winter of 2021–2022 [13,23,71]. Seroprevalence was also lower in our study compared with early 2021 (13% versus 40% [72]) and decreased significantly in 2024–2025. Given that transmission from humans may be a primary mechanism for deer exposure, this trend towards fewer deer infections could result from lower virus availability in human populations relative to during the pandemic [73] and higher human antibody prevalence from vaccination and previous exposure [74,75].

Urban sprawl is expected to increase frequencies and types of interactions between wildlife and humans, potentially resulting in more zoonotic disease transmission [76,77]. Disease emergence surveillance at this interface is therefore increasingly important to public and animal health. The formation and maintenance of active surveillance programmes, such as the one that led to this study's insights, are therefore warranted. Particularly when combined with concurrent studies of human behaviour [78], these programmes provide a foundation for early warning and risk assessment of pathogen transmission across human–animal interfaces.

## Supplementary Material



## Data Availability

R code, as well as health data and derived movement data, are available from the Dryad Digital Repository (https://doi.org/10.5061/dryad.zcrjdfnsc). Supplementary material is available online [79].

## Declaration of AI use

We have used AI-assisted technologies in creating this article. AI primarily assisted with this work in a similar capacity to an interactive search engine, most notably in debugging code and clarifying statistical concepts. Example prompts and responses are given in the AI supplement document under the heading Usage of ChatGPT to help with coding. Given that this was an iterative, exploratory process and often not directly relevant to the final product, the examples given are not exhaustive. AI was also used sparingly as an editor to improve the clarity of parts of the Material and methods, Results and Discussion, but initial and final writing was done by humans. The prompts and responses that were used in writing are listed in the AI supplement under the heading ChatGPT in writing. We used ChatGPT 5.0 (occurred 10 January–7 August (version 4o), and 8 August–October 2025 (version 5)) and Claude (occurred 1 May–10 June 2026 (Sonnet 4.6)).
