# Supplementary material for: SARS-CoV-2 exposure in deer increases with use of human-modified habitat
Source: Biol Lett. 2026 Nov 4;22(11):20260410. doi: 10.1098/rsbl.2026.0410 (PMC13730802; doi:10.1098/rsbl.2026.0410)
Supplement: Supplementary file 1 [file rsbl.2026.0410.zip › rsbl.2026.0410/rsbl20260410_si_001.docx]

**Supplementary Information**

**Title**:

Supplementary Information for: “Exploring how movement patterns and human encounters influence SARS-CoV-2 seropositivity in deer”

**Authors**:

Tyler J Garwood^1^, Jeremy Alder^2^, Sara Hathaway^2^, George Wittemyer^2^, Guillaume Bastille-Rousseau^3^, Tadao Kishimoto^3^, Michael Egan^3^, Travis Gallo^4^, Jennifer M. Mullinax^4^, Amira A. Roess^5^, Taylor Anderson^6^, Tiffany M. Wolf^7^, James D. Forester^8^, Jennifer L. Malmberg^9^, Marnee Roundtree^10^, Georgia C. Titcomb^2^, Rebecca M. Windell^2^, Maria A. Diuk-Wasser^11^, Laura D. Plimpton^11^, Meredith C. VanAcker^12^, Daniel M. Grove^13^, Justin R. Kosiewska^13^, Dailee L. Metts^13^, Cameron Mitchell^13^, Lisa I. Muller^13^, Mark Q. Wilber^13^, Jacob D. Wyrick^13^, Kezia R. Manlove^14^, Megan Morrison^14^, Aung N. Chan^14^, Jennifer Høy-Petersen^15^, Roderick B. Gagne^15^, W. David Walter^16^, Alec Baker^16^, Jeffrey C. Chandler^9^, Grete Wilson-Henjum^9^, Kim M. Pepin^9^, and Meggan E. Craft^1^

**Affiliations**:

^1^ Department of Ecology, Evolution, and Behavior, University of Minnesota, 1987 Upper Buford Circle, St. Paul, MN 55108, USA

^2^ Department of Fish, Wildlife and Conservation Biology, Colorado State University, 1474 Campus Delivery, Fort Collins, CO 80523, USA

^3^ Center for Wildlife Sustainability Research, Southern Illinois University, 1125 Lincoln Drive, Carbondale, IL 62901, USA

^4^ Department of Environmental Sciences and Technology, University of Maryland, College Park, MD 20742, USA

^5^ Department of Global and Community Health, George Mason University, 4400 University Drive, MS 5B7, Fairfax, Virginia 22030, USA

^6^ Department of Geography and Geoinformation Science, George Mason University, Exploratory Hall, MSN 6C3, Fairfax, Virginia 22030, USA

^7^ Department of Veterinary Population Medicine, University of Minnesota, 1365 Gortner Ave, Saint Paul, MN 55108, USA

^8^ Department of Fisheries, Wildlife, and Conservation Biology, University of Minnesota, 2003 Upper Buford Circle, Suite 135, St. Paul, MN 55108, USA

^9^ National Wildlife Research Center, Wildlife Services, Animal and Plant Health Inspection Service, United States Department of Agriculture, 4101 Laporte Ave, Fort Collins, CO 80521, USA

^10^ Nebraska Game and Parks Commission, 299 Husker Road, Alliance, NE 69301, USA

^11^ Department of Ecology, Evolution, and Environmental Biology, Columbia University, 1200 Amsterdam Ave, New York, NY 10027, USA

^12^ Department of Evolution, Ecology, and Organismal Biology, University of California - Riverside, 900 University Ave, Riverside, CA 92521, USA

^13^ School of Natural Resources, University of Tennessee Institute of Agriculture, 2431 Joe Johnson Dr, Knoxville, TN 37996, USA

^14^ Department of Wildland Resources and Ecology Center, Utah State University, 5230 Old Main Hill, Logan, UT 84322, USA

^15^ Department of Pathobiology, Wildlife Futures Program, University of Pennsylvania School of Veterinary Medicine, New Bolton Center, Kennett Square, PA 19348 USA

^17^ Pennsylvania Cooperative Fish and Wildlife Research Unit, The Pennsylvania State University, 413 Forest Resources Bldg., University Park, PA 16802, USA

Table S1: A summary of the sample size of individual deer used in analysis by site, broken down by deer demographic traits.

| **Site** | **State** | **Sample Size** | **Female** | **Male** | **Juvenile** | **Adult** | **White-tailed Deer** | **Mule Deer** |
| --- | --- | --- | --- | --- | --- | --- | --- | --- |
| Ames UT/REC | Tennessee | 49 | 36 | 13 | 0 | 49 | 49 | 0 |
| Carver Park Reserve | Minnesota | 42 | 34 | 8 | 16 | 26 | 42 | 0 |
| DMA2 | Pennsylvania | 78 | 56 | 22 | 21 | 57 | 78 | 0 |
| Elm Creek Park Reserve | Minnesota | 46 | 36 | 10 | 15 | 31 | 46 | 0 |
| La Sals | Utah | 106 | 84 | 22 | 6 | 100 | 0 | 106 |
| Lone Oaks | Tennessee | 10 | 6 | 4 | 0 | 10 | 10 | 0 |
| Nebo | Utah | 133 | 111 | 22 | 23 | 110 | 0 | 133 |
| Oquirrh / Stansbury | Utah | 62 | 62 | 0 | 7 | 55 | 0 | 62 |
| Pine Valley | Utah | 59 | 40 | 19 | 26 | 33 | 0 | 59 |
| Platte | Colorado | 21 | 17 | 4 | 7 | 14 | 17 | 4 |
| Poudre | Colorado | 89 | 49 | 40 | 16 | 73 | 35 | 54 |
| Range Creek | Utah | 103 | 103 | 0 | 10 | 93 | 0 | 103 |
| Southern Illinois University Campus | Illinois | 3 | 1 | 2 | 2 | 1 | 3 | 0 |
| San Juan | Utah | 124 | 122 | 2 | 15 | 109 | 0 | 124 |
| Scottsbluff | Nebraska | 53 | 43 | 10 | 8 | 45 | 0 | 53 |
| Shakopee | Minnesota | 24 | 18 | 6 | 10 | 14 | 24 | 0 |
| Shelbyville | Illinois | 40 | 24 | 16 | 18 | 22 | 40 | 0 |
| Staten Island | New York | 44 | 29 | 15 | 0 | 44 | 44 | 0 |
| Touch of Nature | Illinois | 27 | 19 | 8 | 6 | 21 | 27 | 0 |
| Treasure Lake | Pennsylvania | 38 | 30 | 8 | 0 | 38 | 38 | 0 |
| Totals |  | 1151 | 920 | 231 | 206 | 945 | 453 | 698 |

Table S2: Number of animals with a given dominant fix interval across our study sites.

| **Site** | **State** | **15 min** | **30 min** | **60 min** | **90 min** | **120 min** | **210 min** | **270 min** |
| --- | --- | --- | --- | --- | --- | --- | --- | --- |
| Platte | Colorado | 19 | 2 | 0 | 0 | 0 | 0 | 0 |
| Poudre | Colorado | 78 | 10 | 1 | 0 | 0 | 0 | 0 |
| Southern Illinois University Campus | Illinois | 0 | 3 | 0 | 0 | 0 | 0 | 0 |
| Shelbyville | Illinois | 0 | 40 | 0 | 0 | 0 | 0 | 0 |
| Touch of Nature | Illinois | 0 | 27 | 0 | 0 | 0 | 0 | 0 |
| Carver Park Reserve | Minnesota | 0 | 25 | 0 | 17 | 0 | 0 | 0 |
| Elm Creek Park Reserve | Minnesota | 0 | 34 | 0 | 12 | 0 | 0 | 0 |
| Shakopee | Minnesota | 0 | 10 | 0 | 14 | 0 | 0 | 0 |
| Scottsbluff | Nebraska | 1 | 0 | 52 | 0 | 0 | 0 | 0 |
| Staten Island | New York | 0 | 44 | 0 | 0 | 0 | 0 | 0 |
| DMA2 | Pennsylvania | 3 | 54 | 0 | 0 | 0 | 12 | 9 |
| Treasure Lake | Pennsylvania | 0 | 38 | 0 | 0 | 0 | 0 | 0 |
| Ames UT/REC | Tennessee | 0 | 49 | 0 | 0 | 0 | 0 | 0 |
| Lone Oaks | Tennessee | 0 | 10 | 0 | 0 | 0 | 0 | 0 |
| La Sals | Utah | 0 | 40 | 4 | 0 | 62 | 0 | 0 |
| Nebo | Utah | 0 | 70 | 0 | 0 | 63 | 0 | 0 |
| Oquirrh / Stansbury | Utah | 0 | 37 | 0 | 0 | 25 | 0 | 0 |
| Pine Valley | Utah | 0 | 23 | 3 | 0 | 33 | 0 | 0 |
| Range Creek | Utah | 0 | 16 | 0 | 0 | 87 | 0 | 0 |
| San Juan | Utah | 0 | 70 | 6 | 0 | 48 | 0 | 0 |
|  | **Totals** | 101 | 602 | 66 | 43 | 318 | 12 | 9 |

Table S3: Pearson’s correlations between numeric covariates considered for modeling.

| **Variable** | Average human footprint index | Proportion of locations in deer habitat | Proportion of locations in human development | Proportion of locations in forest | Median speed | Standardized average human population density | Standardized average maximum 6 day range | Standardized human SARS-CoV-2 wastewater concentration |
| --- | --- | --- | --- | --- | --- | --- | --- | --- |
| Average human footprint index | NA | -0.11 | 0.56 | -0.07 | -0.04 | 0.18 | -0.02 | 0.01 |
| Proportion of locations in deer habitat |  | NA | 0.01 | 0.29 | 0.29 | 0.03 | -0.04 | -0.01 |
| Proportion of locations in human development |  |  | NA | 0.03 | -0.09 | 0.22 | -0.03 | -0.03 |
| Proportion of locations in forest |  |  |  | NA | -0.24 | -0.08 | 0.02 | 0.03 |
| Median speed |  |  |  |  | NA | 0.06 | 0.41 | -0.12 |
| Standardized average human population density |  |  |  |  |  | NA | -0.02 | 0.02 |
| Standardized average maximum 6 day range |  |  |  |  |  |  | NA | -0.04 |
| Standardized average human SARS-CoV-2 wastewater concentration |  |  |  |  |  |  |  | NA |

Table S4: Results from a leave-one-out validation by site. All effect sizes and directions are stable and therefore unlikely to be driven by a single site.

| **Effect** | **Mean** | **Standard Deviation** | **Minimum** | **Maximum** |
| --- | --- | --- | --- | --- |
| Age Class: Juvenile | -1.12 | 0.10 | -1.38 | -0.97 |
| Average human footprint index | 0.07 | 0.00 | 0.05 | 0.08 |
| Sex: Male | -0.13 | 0.05 | -0.22 | -0.01 |
| Standardized average human SARS-CoV-2 wastewater concentration | 0.97 | 0.15 | 0.58 | 1.31 |
| Species: White-tailed deer | -0.28 | 0.03 | -0.37 | -0.23 |
| Biological year: 2023-24 | -0.48 | 0.06 | -0.58 | -0.30 |
| Biological year: 2024-25 | -1.06 | 0.17 | -1.61 | -0.70 |

Figure S1: A depiction of when each test in the study occurred. Note that tests were often conducted en masse in a site at the same time in a given year due to good weather conditions for capture or helicopter availability.

Figure S2: The log-odds estimates for the coefficients in the top model (seropositivity ~ biological year + age class + sex + species + av. HFI + st. av. human wastewater concentration + site) using two-, four-, and six-month post-capture time window for movement / space use covariate extraction. Coefficients are consistent across time windows, indicating that results are not highly sensitive to the time window used.

Figure S3: The covariate coefficient values are similar across models.

Figure S4: A plot of the AICc values for top model (seropositivity ~ biological year + age class + sex + species + av. HFI + st. av. human wastewater concentration + site) using two- (AICc = 761), four- (762), and six- month (765) post-capture time window for movement / space use covariate extraction. Only the subset of individuals that were in all time periods were used to calculate AICc when determining the apprpriate time period (n= 1007), but coefficients in the main text are given based on the entire 2 month dataset (n = 1115).

**Supplementary text:**

**IACUC Protocol numbers:**
Colorado State University, CO: #3872 and #5002

Southern Illinois University, IL: #21-028

University of Minnesota, MN: #2209-40440A

Columbia University, NY: #AC-AABT8667

Pennsylvania State University, PA: #202202225

University of Tennessee Knoxville, TN: #2850-1021

Utah State University, UT: #13045).

**Sample handling, testing procedures, and case definitions**

Swabs were placed immediately in PrimeStore® Molecular Transport Media (Thermo Fisher Scientific Inc., Waltham, MA, USA), refrigerated or frozen, and tested for SARS-CoV-2 viral RNA via real-time polymerase chain reaction with reverse transcription at USDA National Wildlife Research Center’s Wildlife Disease Diagnostics Laboratory (WDDL).^1-2^ Blood samples were collected via both syringe/needle and Nobuto strips. Blood was centrifuged to serum (syringe/needle) or antibodies were eluted (Nobuto strips) for surrogate virus neutralization tests (sVNT: cPASStm SARS-CoV-2, Genscript, Piscataway, NJ, USA) at WDDL.^3^ “Wildtype” sVNT tests are designed to detect all non-omicron variants.

**K-steps normalization approach**

To illustrate the k steps normalization approach, assume we have an animal with 30 minute interval data. Because there are nine 30-minute intervals in 270 minutes, we assign k = 9. We then calculate the displacement (meters) between locations 1 and 10 (i.e. 1+k), then 2 and 11 (2+k), up to k - 9, for that individual and divide each displacement by 270 min for speed. Similarly, we calculate the turning angle between points 1, 10, and 19, (2, 11, and 20, etc). We then summarize these calculations for this individual by calculating median speed (meters / hour). In practice, we retained intervals where the elapsed time was within 25% of 270 minutes and divided displacement by the actual time elapsed between fix number + k for speed. This approach allows for estimates of these metrics on a similar scale without discarding location data collected at more frequent intervals.

**Variable extraction process**

For **deer demographic traits:**

1. **Age:** Determined at capture via tooth wear, assigned to either Juvenile (<1.5) or Adult (>1.5) based on estimated age.
2. **Sex:** Determined at capture.
3. **Species**: determined at capture.

For **movement behaviors:**

1. **Standardized average maximum 6 day range:** Calculated maximum distances between an individual deer’s locations for each 6 day period, and took the average across the weeks that deer was observed. Standardized the average maximum distance between locations by site with z-scores.
2. **Median speed**: Standardized locations to ~270 min intervals with k-steps normalization, and divided displacement by time elapsed to calculate speed for each interval. Summarized speed for each individual by the median speed across intervals.

For **t**i**me spent near humans or human modified landscapes:**

1. **Standardized average human population density**: Extracted human population density at each point. Calculated average for each deer. Standardized with z-score.
2. **Proportion of locations in human development**: Extracted cover type at each point. Calculated proportion of points that are in any "development" category (NLCD classes 21-24) for each deer.
3. **Average human footprint index**: Extracted human footprint index value at each point. Calculate average for each deer.

For **t**i**me spent near humans or human modified landscapes**:

1. **Standardized average SARS-CoV-2 human wastewater concentration:** Calculated the average human wastewater concentration (average gene copies/person/day) over the previous 13 months in the county that the deer’s site was in. When no county data was available, we assumed data from the nearest county with data and similar human demographic traits was a suitable proxy. Standardized with z-score.

For **time spent in habitat that is suitable for deer**:

1. **Proportion of locations in deer habitat**: Combine white-tailed and mule deer species GAP maps and reclassify such that pixels with suitable habitat for 1 species are "1", and suitable for both species are "2". Extract the value type at each deer point. Calculate proportion of points that have a value of 1or 2 for each deer
2. **Proportion of locations in forest (used in interaction with Species)**: Proportion of points in forest: Extract cover type at each point. Calculate proportion of points that are in any "forest" category (classes 41-43) for each deer

For **sampling design,**

1. **Biological yea**r: Based on when the deer was caught within a biological deer year, which was defined June 1-May 31 to align with fawning (i.e. new wave of susceptible individuals).

**References**

1. Bevins SN, Chipman RB, Beckerman SF, Bergman DL, Collins DT, Deliberto TJ, Eckery JP, Ellis JW, Gosser AL, Heale JD, et al. (2023). SARS-CoV-2 occurrence in white-tailed deer throughout their range in the conterminous United States. *bioRxiv*. 2023;p. 2023.04.14.533542. https://doi.org/10.1101/2023.04.14.533542
2. Feng A, Bevins S., Chandler J, DeLiberto TJ, Ghai R, Lantz K, Lenoch J, Retchless A, Shriner S, Tang CY, et al. Transmission of SARS-CoV-2 in free-ranging white-tailed deer in the United States. *Nat. Commun.* 2023;*14*(1):4078. https://doi.org/10.1038/s41467-023-39782-x
3. Hewitt J, Wilson-Henjum G, Chandler JC, Phillips AT, Diel DG, Walter WD, Baker A, Høy-Petersen J, Bastille-Rousseau G., Kishimoto T, et al. Evaluation of SARS-CoV-2 antibody detection methods for wild Cervidae. *Prev. Vet. Med.* 2025;*41*:106522. https://doi.org/10.1016/j.prevetmed.2025.106522
